# Care seeking pathways of older adults with hip fracture in India: exploratory study protocol

**DOI:** 10.1186/s12939-015-0220-9

**Published:** 2015-11-14

**Authors:** Abha Tewari, Kirti Sundar Sahu, Lalit Yadav, Sanghamitra Pati, Srinivas Nallala, Premilla Webster, Robyn Norton, Santosh Rath

**Affiliations:** The George Institute for Global Health, New Delhi, India; Indian Institute of Public Health, Public Health Foundation of India, Bhubaneshwar, India; Nuffield Department of Population Health, University of Oxford, Oxford, UK; Nuffield Department of Population Health, The George Institute for Global Health, University of Oxford, Oxford, UK; The George Institute for Global Health, Sydney Medical School, University of Sydney, 34 Broad Street, Sydney, OX1 3BD Australia

**Keywords:** Care seeking, Behavior, Hip fracture, Adult, India, Qualitative, Exploratory, Decision, Delay, Protocol

## Abstract

**Background:**

The incidence of hip fractures in older adults in India is likely to increase dramatically in the coming decades as a result of an aging population and increasing life expectancy. Currently, more than 600,000 adults over 60 years of age suffer a hip fracture annually in India. This paper outlines a protocol for a qualitative study investigating the care seeking behavior of older adults with hip fractures: to determine the processes in decision making, identify causes for delay in obtaining care, and identify potential barriers and facilitators to seeking appropriate care in time.

**Methods and design:**

The planned study will consider Odisha, an eastern state in India with limited health care facility, as a suitable case study. It is proposed to conduct 30 in-depth interviews in two administrative districts of Odisha. The participants will be patient and their carers in seven health facilities- four public hospitals, two private hospitals and one traditional bone-setting facility. The study relies on a purposive sampling strategy. Ethics permission will be sought from each participating institution and participants. The participants will be adults aged 50 years or older of both sexes arriving at the recruiting centers with a history of fall or injury, pain in the hip region and inability to walk and X-ray confirmed diagnosis of proximal femoral fracture and their primary carer. Trained qualitative research team will conduct these interviews. A thematic framework approach will be used to analyze the data using NVivo 9 software.

The data collected from the interviews will be analysed to explore the cause of the hip fracture, events following the injury, the experiences of patients from the time of sustaining the injury, pain relief measures, decision to seek care, understanding of the urgency for treatment, causes for delay in receiving treatment, funding sources, cost liabilities for the family, financing mechanisms for out of pocket expenditure and the burden for caring.

**Discussion:**

The findings of this study will provide an increased understanding of the care seeking behaviors of older adults with hip fracture, and inform contextually appropriate changes in healthcare program and policy aimed at improving health outcomes.

## Background

The incidence of hip fractures in older adults in India is likely to increase dramatically in the coming decades as a result of an aging population and increasing life expectancy. By 2020, it is estimated that India will have a population of 1305 million of which 82.2 million (6.3 %) will be over 65 years [1]. Conservative estimates suggest that at present more than 600,000 adults over 60 years of age suffer a hip fracture annually in India [2]. The numbers with hip fractures are likely to be greater, as women over 50 years of age in India are susceptible to fractures due to osteoporosis; [3] otherwise known as fragility fractures. Hip fractures in older adults are common injuries in high-income countries and 30 % of them die within 1 year of hip fracture injury. Nearly half these older people after a hip fracture injury do not return to their usual place of residence and require assistance for activities of daily living. Various studies have established that delay in hip fracture surgery increases mortality [4] and the UK National Hip Fracture Database (NHFD) audit, including more than 250,000 patients, has demonstrated improved outcomes by early surgery [5]. The best practice guidelines for managing older adults with hip fracture are: fast track admission to a suitable treatment center, collaborative orthogeriatrician care, surgery within 48 h of injury, early post-operative mobilization, and medication for osteoporosis and fall prevention education. These practices have reduced peri-operative complications and mortality and facilitated earlier discharge from hospital and improved quality of life (QoL) in terms of physical,mental and social well being [6] In the UK, the NHFD audit has demonstrated that early surgery within 48 h is key to improving QoL of older people with hip fractures. Consequently, adopting a similar approach in India has the potential to prevent unnecessary deaths and improve QoL of an overwhelming number of older adults expected to suffer a hip fracture in India [7].

As time to surgery from a hip fracture is key to improving outcomes, the speed of arrival at the surgical centre following the fracture is crucial. Various factors contribute to delays in reaching treatment centres and receiving appropriate care. The framework of the three-delay model, developed for analysing barriers to emergency caesarean section, identified decision making to seek care as the first cause for delay, transportation as the second cause for delay and the third delay is in receiving care within the hospital [8]. This model provides a generic framework to group events and activities occurring at home following a health condition and then during transportation to a care centre in countries with fragmented health systems and finally delays in receiving care in hospital due to factors related to health system capacity and organisation.

The decision-making processes to seek care are influenced by a multitude of factors. Cultural and religious beliefs, faith, limited societal knowledge, inequities of age and gender, poor access to health facilities and financial barriers drives care-seeking behavior [9]. Faith in traditional bone-setters and osteopaths influences care seeking behavior for musculoskeletal conditions in India [10]. Family members often opt for traditional treatment in children and proceed to professional care if symptoms are unresolved [11]. Lack faith in the medical skills of the staff and machinery in secondary care in rural China, leads to overcrowding of tertiary care [12].

Family members with financial and caring responsibilities often take the major role in deciding treatment options and may cause age and gender inequities [13].

The supply side of care, cost and ease of access significantly influence care-seeking behavior. Surgical facilities for fracture treatment in India are mostly limited to tertiary care hospitals in urban areas and at great distances. In Kerala, the state with the best healthcare standards in India, elderly adults mostly seek private care [14] whereas in West Bengal, most attend public health care facilities [15]. There is evidence that access to surgical care is less in low and middle-income countries (LMIC) and those who are poor and, in particular women, tend to be affected more with added risk of adverse outcomes [16].

Understanding the decision-making processes to seek care, which contributes to the first delay, will identify processes and attitudes that are barriers to implementation of best practices in the management of hip fractures in India. The proposed study will be conducted in Odisha, an eastern state in India with a population of 43 million and more than 9 % over 60 years of age. The Public Health System in Orissa comprises various levels of facilities in each of the 30 adminstartive district, and tertiary care facilities for referral [17].

Each district hospital (DH) serves a population of approximately 1.3–2 million population. Few of these DH are equipped for orthopaedic surgery and majority of patients seek care in private hospitals, mostly in urban areas. The poor can afford care only at public tertiary care facilities, which are overcrowded and at a great distance from their homes. There are no data from Odisha or India on the care-seeking pattern of older adults, as to when, why and how they choose a treatment facility. Our proposed study aims to document the present care seeking pathways for older adults with hip fractures in Odisha.

## Aims and objectives

The aim of the study is to provide evidence to strengthen the health systems for the delivery of evidence based care for older adults with hip fractures in Odisha. The specific objectives of the study are to explore the care seeking behavior of older adults with hip fractures: to determine the processes in decision making, identify causes for delay in obtaining care, and identify potential barriers and facilitators to seeking appropriate care in time.

## Methods and design

### Study design

**Fig. 1:**
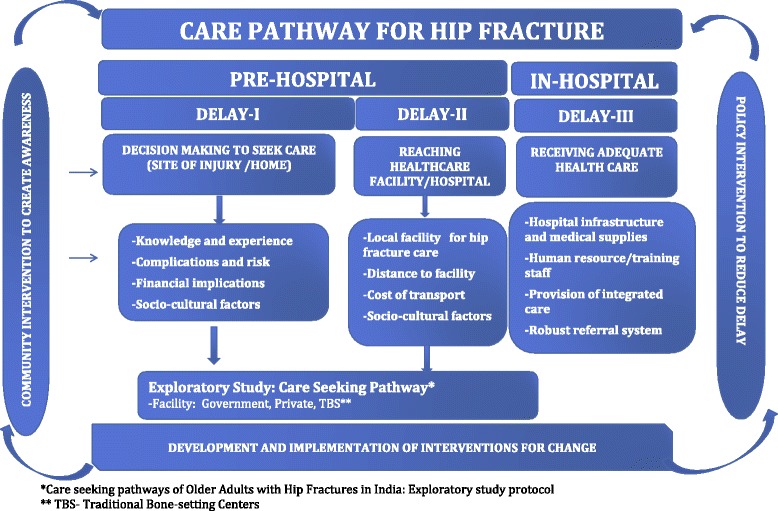
Proposed conceptual framework of care pathway for hip fracture management

This is an exploratory study using qualitative methods. Qualitative research takes a detailed approach to the phenomena under the study in order to understand it more thoroughly. Therefore, in-depth interviews are a suitable method for this approach because of their emphasis on people’s lived experience. They are considered to be well suited for locating the meanings that people place on the events, processes, and structures of their lives and their perceptions, presuppositions and assumptions [18]. Moreover, a study concerning patients/carers’ perspectives requires a qualitative approach to enhance understanding of the context, personal experiences and interpretations of participant. In this study qualitative data will seek to understand the events leading to the fracture, decision making processes in the family to seek care, transfer to a facility, inter-hospital referral or self -referral till patient’s admission into a facility for definitive fracture management (Fig. 1).

### Study setting

The study relies on a purposive sampling strategy. The study will be carried out in Khordha and Cuttack administrative districts of Odisha. Hip fractures arriving at Khorda District Head quarter Hospital (DHH) are often referred to the nearest non-teaching tertiary care facility at Capital Hospital (CH) in Bhubaneswar or the government SCB Medical College Hospital (SCBMCH), Cuttack. Three public hospitals (DHH, CH & SCBMCH) and two private hospital in a geographical area, Kalinga Institute of medical sciences (KIMS), Bhubaneswar, Ashwini Hospital & Trauma Centre, Cuttack have been identified as participating sites for the study. To identify factors influencing decisions to seek treatment with traditional osteopaths, hip fracture patients seeking care with the regional osteopath at Kalupada will also be included in the study.

It is proposed to conduct 30 in-depth interviews with patients and carers in different healthcare facilities. Purposively,seven institutions;four public hospitals and two private hospitals and one traditional bone-setting center have been identified as sites for the study. Ethics permission will be sought from each participating hospital. The patients will be recruited purposively after they are admitted to the care facilities. The clinicians will identify patients as per the inclusion criteria of the study, introduce researcher to the patient and facilitate consent procedure.

#### Participants

The inclusion criteria for the study are adults aged 50 years or older of both sexes arriving at the recruiting centers with a history of fall or injury, pain in the hip region and inability to walk and, X-ray confirmed diagnosis of proximal femoral fracture, also known as a hip fracture. Patients with pathological fractures and terminal malignancies will be excluded. The person with the hip fracture will be the informant for the study and the primary carer will be requested for additional information. Participants will be provided with verbal and written information in local language on the aims and methods of the study and consent will be obtained from the participants prior to the interview. They will also be informed that all data collected will be stored safely and only used by researchers, maintaining confidentiality.

#### Data collection and analysis

In-depth, qualitative interviews will be carried out by the research team with the assistance of an interview guide (Appendix 1). Literature review has been done prior formulating the questions for the interview guide. These questions will be flexible and allow prompts to be used [19]. The interview guide will be translated from English to Odia and back translated from Odia to English by trained translators. In order to maintain the quality and accuracy of the translation these document will be validated by the research team members. The guide will be pilot tested with participants not included in the study and revised accordingly.

The interview will explore the experiences of patients from the time of sustaining the injury, exploring the cause of the hip fracture, events following the injury, the experience of patients from the time of sustaining the injury, pain relief measures, decision to seek care, understanding of the urgency for treatment, causes for delay in receiving treatment, funding sources, cost liabilities for the family, financing mechanism for out of pocket expenditure and the burden for caring. A questionnaire will be developed using categories identified from the data collected from the interviews.

**Fig. 2:**
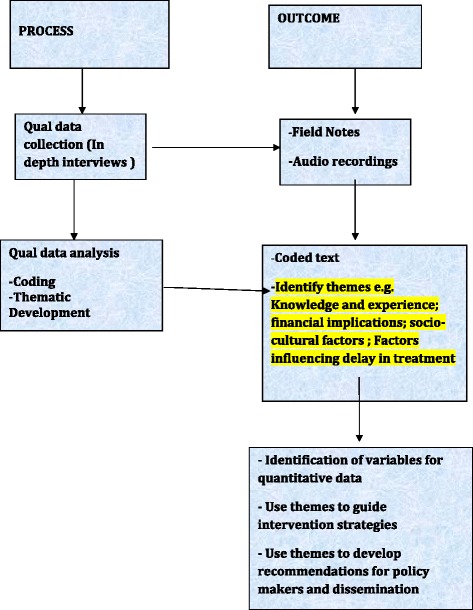
Overview of process and outcome

Thirty patients with hip fracture will be interviewed within the care setting as per convenience of the participants. Trained qualitative research team members will conduct these interviews and each team will comprise of a moderator and note-taker. Field notes will be taken by the note-taker at all occasions. Odia transcriptions of all interviews and then translate to English. All the interviews will be audio-recorded (with participants’ consent) and will be transcribed verbatim and then translated to English. Data will be analysed using conventional qualitative methods that seek to identify themes which are relevant both across and in cases [20]. All transcripts/data will be reviewed by two members of the research team to identify the recurrent themes to minimize the risk of subjectivity and established validity. The research team will familiarize with the responses by going through the transcribed data repeatedly. During the familiarization process, broad thematic areas that emerge from the data will be identified. Thematic analysis provides a concise, coherent, logical, non repetitive and interesting account of the sequence of events. The next step will be to assign data to different themes, which is called coding. Then the responses based on the codes will be grouped under each theme and these are called thematic charts. Based on these charts, interpretation of the data will be done and report will be presented under different theme. During the analysis the English version will be controlled and compared with the Odia transcriptions in order to ensure inclusion of all relevant context and local matters in the analysis [21]. The transcriptions will be coded independently by two researchers involved in the data collection. The coding and thematic analysis will be done by Using NVIVO 9, qualitative data analysis software. The findings will be presented in themes and subthemes. A theme is an expression or context being defined as important by the researchers and captures a significant meaning in relation to the research questions and aims (Fig. 2).

### Possible outcome of the analysis

The data collected from the interviews will be analyzed to identify factors influencing decision making in the family to seek care for older adults with hip fracture, the choice of care facility, influence of equity and gender in receiving appropriate care in time. The study will inform on the following:

- Patient’s experience following hip fracture and awareness of the consequences of the injury
- Factors influencing delay in treatment: carer involvement in decision making, household finance in care seeking decisions, availability and access to treatment, transport, and availability of surgical care in local hospital
- Socio-cultural factors: Belief in a particular discipline of treatment, preference for the treatment and patient satisfaction

## Discussion 

This exploratory study will provide an in-depth understanding of the processes involved in seeking care for older adults with hip fracture in the recruitment area. Qualitative research generally takes more time to collect the data when compared to quantitative research. In addition, information collected through qualitative methods is contextual and may not be generalizable in other settings. Therefore, in order to maximize the benefits of interview, data quantification is necessary. A structured questionnaire will be developed using categories identified from the data collected through in-depth interviews [18]. The questionnaire for care seeking behavior will enable rapid survey to identify factors that are quantitatively relevant and contextually pertinent, and thereby inform evidence supported intervention strategies to improve management of older adults with hip fractures [22]. It is hoped that this methodology will find wider application to understand care seeking behavior for other clinical conditions and inform contextually appropriate changes in healthcare program and policy.

### Limitation of the study

The proposed study conducted in two purposively selected district portrayed the situation for hip fracture care in Odisha state. As socio economic condition and health care delivery system are not uniform throughout the country, the result cannot be generalized. Such studies are therefore needed in different parts of India and other developing countries.

### Ethics and dissemination

The study has been evaluated and approved by the ethics committee of the Center for Disease Control (CCDC, New Delhi) (IEC/04/2014 dated 1.10.2014) and Government of Odisha (224/SHRMU dated 31.07.2014). The findings from this study will be disseminated through peer-reviewed publications and national & international conferences. Articles will also be submitted to other types of media to increase the awareness on the issue.

## Appendix

### Interview Schedule for Patients and/or Carers

1. Name of respondent:
2. Age of respondent:
3. Gender:
4. Education:
5. Hospital Name & Address
6. Distance from your residence:
7. Name of interviewer:
8. Name of note-taker:
9. Date of interview:
10. Interview start time ............end time:
11. Please tell us about the purpose of your hospital visit?
  *Probe: specific injury or event, site of injury, time, place etc.*
12. Please elaborate your personal experience/feelings during the time of injury/fall?
  *Probe: Symptoms related to discomfort, pain, bleeding, lost consciousness etc.*
13. Could you please describe the events that have happened immediately after the injury.
  - *Did you receive any help or support at that time? If yes, who supported you?*
  - *What was their initial action?*
14. Which of your family member/s supported you at that time? What was their initial reaction towards this event?
  *Probe: number of family members, decision maker in the family*
15. When did you decided to seek help from health care centre/hospital and how?
  *Probe: first point of contact, who accompanied you, mode of transport, time took to reach the centre, any preliminary treatment*
16. Are you aware of the existing health care centres/hospitals in your area? Give details
17. In your opinion what are other hospitals in your area which can provide similar kind of treatment/care? What are the reasons for choosing this hospital?
  *Probe: Facility, distance, cost, quality etc.*
18. How was your experience at the health care centre/hospital?
  *Probe: registration related, who attended you first, waiting period, any first aid given*
19. What treatment was given at the centre/hospital?
  *Probe: treatment, duration of stay,who paid for the treatment, referral to other/higher centre, reason for refer?*
20. What was the second point/subsequent point of contact with health care facility. Please give details
  *Probe: treatment, duration of stay, who paid for the treatment?*
21. Did you have any fracture before, If yes, could you tell us about it in detail
  - *Where was the fracture?*
  - *When was the fracture?*
  - *What did you do?*
  - *Where did you seek treatment?*
  - *Did you go for physiotherapy/alternative treatment, was there any residual pain/activity limitation post fracture/injury?*
  - *Do you have any other health problem/concerns?*
22. Any other suggestion?
